# Roles of pectin in biomass yield and processing for biofuels

**DOI:** 10.3389/fpls.2013.00067

**Published:** 2013-03-27

**Authors:** Chaowen Xiao, Charles T. Anderson

**Affiliations:** ^1^Department of Biology, The Pennsylvania State UniversityUniversity Park, PA, USA; ^2^Center for Lignocellulose Structure and Formation, The Pennsylvania State UniversityUniversity Park, PA, USA

**Keywords:** pectin, cell wall, cell adhesion, gelling, biomass, lignocellulosic biofuel

## Abstract

Pectin is a component of the cell walls of plants that is composed of acidic sugar-containing backbones with neutral sugar-containing side chains. It functions in cell adhesion and wall hydration, and pectin crosslinking influences wall porosity and plant morphogenesis. Despite its low abundance in the secondary cell walls that make up the majority of lignocellulosic biomass, recent results have indicated that pectin influences secondary wall formation in addition to its roles in primary wall biosynthesis and modification. This mini-review will examine these and other recent results in the context of biomass yield and digestibility and discuss how these traits might be enhanced by the genetic and molecular modification of pectin. The utility of pectin as a high-value, renewable biomass co-product will also be highlighted.

## INTRODUCTION

In a society with an increasing demand for renewable energy, plant species as diverse as switchgrass, sugarcane, *Miscanthus*, *Jatropha*, poplar, willow, and *Agave* have been put forward as candidates for lignocellulosic feedstocks to produce liquid biofuels with low net greenhouse gas emissions (Carroll and Somerville, 2009; Somerville et al., 2010). However, many challenges and limitations remain for the economical and efficient conversion of biomass to biofuel (Somerville et al., 2010). Two central challenges are the recalcitrance of biomass to degradation by enzymes into its component sugars, and the fact that plant biomass contains many different hexose and pentose monosaccharides, all of which must be converted into useful products in order to capture the full energy content and value of lignocellulosic feedstocks.

Pectin is a major component of the primary cell walls of dicotyledonous plants and is also present in smaller amounts in the secondary walls of dicots and both types of cell walls in monocots (Vogel, 2008). Pectins are highly complex polysaccharides and are composed of at least four subclasses: homogalacturonan (HG), rhamnogalacturonan (RG-I), RG-II, and xylogalacturonan (XGA; Mohnen, 2008). The backbones of HG, RG-II, and XGA consist of α-1,4-linked galacturonic acid (GalA) residues that can be methyl-esterified at the C6 carboxyl group and/or acetylated at O2 or O3, whereas the backbone of RG-I is composed of alternating rhamnose and GalA residues. RG-II possesses complex side chains with at least 12 different types of sugars, RG-I contains structurally diverse side chains consisting mainly of arabinose and galactose along with other sugars, and XGA is essentially HG with added β-1,3-xylosyl side groups (Mohnen, 2008). The synthesis of pectic polysaccharides is estimated to involve at least 67 different enzyme activities, including glycosyltransferases, methyltransferases, and acetyltransferases (Mohnen, 2008; Harholt et al., 2010). Several excellent reviews discuss the details of pectin structure and biosynthesis (Ridley et al., 2001; Willats et al., 2001; Mohnen, 2008; Harholt et al., 2010), which will not be further elaborated upon here.

## ROLES OF PECTIN IN PLANT DEVELOPMENT AND BIOMASS YIELD

Pectin biosynthesis, function, modification, and degradation are involved in several key processes during plant development, including cell wall expansion, cell adhesion, organ formation, cell separation, and phyllotactic patterning (Wolf et al., 2009). Pectin is synthesized in the Golgi apparatus (Moore and Raine, 1988; Moore et al., 1991), which in plants is also the assembly site for glycoproteins, proteoglycans, and other complex polysaccharides (Parsons et al., 2012). Pectin is secreted into the apoplast (the extracellular space that contains the cell wall) in a highly methyl-esterified form (Driouich et al., 2012). One unanswered question is the extent to which pectin and other wall components are sorted during synthesis and trafficking, and whether they first interact with one another before or after secretion.

In the apoplast, pectin can be de-methyl-esterified by the activity of pectin methylesterases (PMEs; Micheli, 2001), and the carboxyl groups of GalA residues can then form intermolecular Ca^2^^+^-mediated crosslinks (Vincken et al., 2003). Additionally, borate diesters can form between the apiose groups of different RG-II molecules, causing them to dimerize (Kobayashi et al., 1996). These crosslinks are generally thought to increase cell wall stiffness: for example, premature de-methyl-esterification restricts hypocotyl elongation in dark-grown *Arabidopsis thaliana* (*Arabidopsis*) seedlings (Derbyshire et al., 2007), and digestion by fungal pectinases or chelation of Ca^2^^+^ by ethylene glycol tetraacetic acid (EGTA) restores the susceptibility of cucumber hypocotyls to the activity of wall-loosening expansins *in vitro* (Zhao et al., 2008). However, recent research has suggested that pectin de-methyl-esterification might also increase its susceptibility to enzymatic degradation, loosening the wall: for instance, pectin de-methyl-esterification facilitates organ primordium initiation in *Arabidopsis* shoot apical meristems (Peaucelle et al., 2011), and overexpression of *PMEI4* delays the growth acceleration of dark-grown *Arabidopsis* hypocotyls (Pelletier et al., 2010). Depending on its consequences, the methyl-esterification status of pectin can thus have complex effects on plant growth (Peaucelle et al., 2012).

Intriguingly, overexpression of a PME inhibitor (PMEI) has resulted in increased biomass in transgenic *Arabidopsis*, as well as slightly increased biomass in transgenic wheat, although the latter difference was not significant (Lionetti et al., 2010). Taken together, the above results suggest that the timing and extent of pectin crosslinking likely influence the growth rate, persistence of expansion, final size, and/or growth robustness of plant tissues, which could in turn influence overall crop yields. Further analysis and manipulation of the links between pectin modification and biomass yield will be an important future research avenue.

## PECTIN AND SECONDARY WALL FORMATION

In addition to its well-established role in primary wall biosynthesis and expansion, some studies have provided evidence for the importance of pectin in secondary cell wall biosynthesis and modification. *PME* genes are expressed in the expanding wood cells of poplar (Siedlecka et al., 2008) and in the stem, phloem, and xylem of southern blue gum (*Eucalyptus globulus*; Goulao et al., 2011). In *E. pilularis*, single-nucleotide polymorphism (SNP) alleles of *PME6* associate with cellulose, lignin, and pulp yield, whereas alleles of *PME7* associate with cellulose, pulp yield, and wood shrinkage (Sexton et al., 2012). Pectin-associated β-1,4-galactans have also been detected in the secondary walls of tension and compression wood (Mellerowicz and Gorshkova, 2012), and upregulation of both pectin-modifying and secondary wall biosynthetic genes has been detected in *Arabidopsis* plants placed under mechanical load (Koizumi et al., 2009). However, these analyses only provide correlative evidence, and genetic, biochemical, and mechanical experiments are required to establish a clearer link between pectin modification and secondary wall formation. In a pioneering study along these lines, *Arabidopsis* mutants lacking *PME35* gene function displayed reduced mechanical integrity in their stem interfascicular fibers (Hongo et al., 2012). Interestingly, all of the above studies highlight pectin-modifying or -degrading genes rather than pectin biosynthetic genes, implying that pectin modification, instead of its synthesis, is an important aspect of secondary wall development.

**FIGURE 1:**
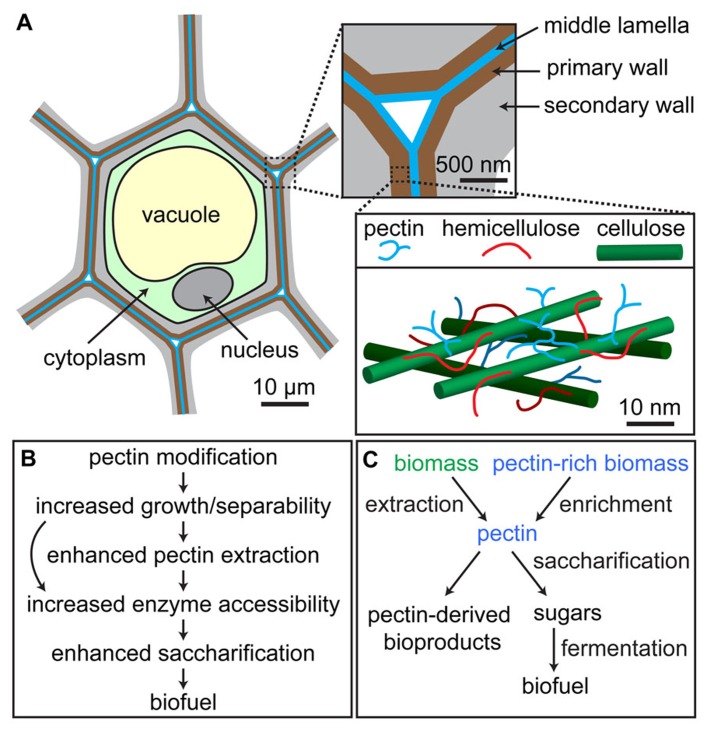
**Location and roles of pectins in biomass.** **(A)** Schematic of plant cell showing arrangement of cell walls; pectin is abundant in the primary walls synthesized by growing cells (brown) and the middle lamella that adheres adjacent cells (blue), but is also present in lower amounts in secondary walls produced after the cessation of growth (gray). Inset at lower right is a simplified model of the primary cell wall showing one possible arrangement of cellulose microfibrils (green), hemicellulose (red), and pectin (blue). **(B)** Pectin-rich biomass can be derived from lignocellulosic feedstocks or naturally pectin-rich plant material, after which it can be processed into pectin-derived high-value bioproducts and/or saccharified and fermented into biofuel. **(C)** Potential positive impacts of pectin modification in bioenergy crop plants on biomass processing. In some cases, pectin modification might allow for the elimination of processing steps, such as pectin extraction (curved arrow in **B**).

Among plant lineages, the presence of RG-II correlates with upright growth, and an increased amount of borate crosslinked RG-II in the cell walls has been postulated to have facilitated the evolution of lignified secondary walls in vascular plants (Matsunaga et al., 2004), implying that pectin might continue to play a role in the early stages of secondary wall deposition. Finally, lignin polymerization, which is an important phase of secondary wall formation in many cell types, has been postulated to initiate in the pectin-rich middle lamella that lies between the walls of adjacent cells (**Figure 1A**), suggesting that there may be a functional connection between these polymers (Westermark et al., 1986). Support for this hypothesis is provided by the finding that addition of pectin affects the *in vitro *dispersion and polymerization of lignin in cellulose networks produced by *Gluconacetobacter xylinus* (Touzel et al., 2003). However, additional evidence will be required to establish a clear and direct connection between pectin biosynthesis and/or modification and secondary wall formation.

## PECTIN AND CELL ADHESION

Intercellular adhesion is a basic feature of plant development and contributes to plant morphogenesis (Knox, 1992). Cell adhesion occurs primarily at the middle lamella, which contains abundant pectins, especially in the reinforcing zones (Jarvis et al., 2003). However, the exact makeup of pectin in the middle lamella is unclear, with some evidence indicating that pectin in this region is mainly composed of RG-I (Moore and Raine, 1988) and other work describing a preponderance of HG (Knox et al., 1990; Willats et al., 1999; Bush et al., 2001). HG chains might also contribute to cell adhesion by crosslinking to other wall components via uronyl esters (Sobry et al., 2005). Antibody labeling of pectin epitopes has provided circumstantial evidence for the function of pectin in cell adhesion (Parker et al., 2001; Sobry et al., 2005), but additional evidence that directly extrapolates the adhesive forces between individual pectin molecules to those between adjacent cells would be informative.

Defective cell adhesion in several mutants has been attributed to insufficient HG–Ca^2^^+^ complexes, branched RG-I polysaccharides, and/or RG-II dimerization (Rhee and Somerville, 1998; Thompson et al., 1999; Shevell et al., 2000; Neumetzler et al., 2012). *Arabidopsis* mutants lacking functional copies of the *QUASIMODO1* (*QUA1*) gene, which encodes the putative GalA transferase GALACTURONOSYLTRANSFERASE 8 (GAUT8), display reduced stature, pectin content, and cell adhesion (Bouton et al., 2002; Leboeuf et al., 2005). Mutants lacking another *Arabidopsis* putative glycosyltransferase, ECTOPICALLY PARTING CELLS 1 (EPC1), also display defective cell adhesion (Singh et al., 2005). However, direct evidence of the role of EPC1 in pectin biosynthesis and cell adhesion is lacking. Mutation in a putative pectin methyltransferase gene, *QUA2*/*TUMOROUS SHOOT DEVELOPMENT2* (*TSD2*), causes reduced cell adhesion and inhibition of shoot development (Krupkova et al., 2007; Mouille et al., 2007). In addition, it has also been shown that polygalacturonases (PGs), which cleave de-methyl-esterified HG, can affect cell adhesion: overexpression of a *PG* gene in apple trees led to altered cell wall adhesion, resulting in abnormal cell separation and plant morphology (Atkinson et al., 2002).

The opposite of cell adhesion, controlled cell separation, occurs in specific tissues and developmental stages in plants and involves the selective degradation of pectin in the middle lamella (Lewis et al., 2006). Artificially controlling cell separation processes might enhance the degradability of engineered biomass feedstocks by increasing the ease with which their cells can be separated by mechanical and/or enzymatic treatments, exposing more surface area to wall-degrading enzymes. However, plants displaying increased cell separability must also maintain growth robustness and disease resistance; thus, inducibly controlled cell separation might be preferable to constitutive activation of this process in future biomass feedstocks (**Figure 1B**).

## PECTIN AND BIOMASS PROCESSING

To efficiently produce biofuels from raw biomass feedstocks, the optimization of methods for pectin extraction and degradation is necessary (Fissore et al., 2011; Min et al., 2011). This is true for two reasons: first, pectin can affect the accessibility of other cell wall components to enzymatic degradation, and second, the sugars contained in pectin itself represent captured photosynthetic energy. In most biomass processing schemes, biomass is first pretreated to disrupt cell wall structure, then saccharified by enzymatic, chemical, or thermal treatment. However, the architectural properties of cell walls, which have been modeled as a cellulose–hemicellulose network embedded in a pectin matrix (**Figure 1A**; Cosgrove, 2000; Dick-Perez et al., 2011), suggest that pectins might mask cellulose and/or hemicellulose (Marcus et al., 2008, 2010), blocking their exposure to degradative enzymes. In fiber hemp processing, pectinase treatment has recently been shown to increase yields of GalA and neutral monosaccharides, and removal of pectin led to increased cell wall surface, improving the accessibility of cellulose to degradative enzymes (Pakarinen et al., 2012). Moreover, modification of pectin by expressing a PG or a PMEI to reduce the total amount of de-methyl-esterified HG in *Arabidopsis*, tobacco, or wheat significantly increased the efficiency of enzymatic saccharification (Lionetti et al., 2010), although PG expression, but not PMEI expression, also led to reduced biomass accumulation in transgenic plants.

The acetyl groups contained in pectin are generally thought to increase biomass recalcitrance by reducing the susceptibility of pectin to enzymatic degradation (Gille and Pauly, 2012). However, surprising results in a recent study (Gou et al., 2012) showed that reduction of pectin acetylation in tobacco by overexpression of a poplar (*Populus trichocarpa*) pectin acetylesterase (*Pt PAE1*) in fact led to lower susceptibility of pectin to degradation, throwing the conventional view into question. Interestingly, the floral styles and filaments of transgenic plants displayed reductions in monosaccharides associated with pectins and increases in monosaccharides associated with cellulose and hemicelluloses (Gou et al., 2012), suggesting that compensatory changes in cell wall composition took place in these tissues. In another study, heterologous expression of a mung bean *PAE* in potato tubers resulted in stiffer tuber tissue, implying that the cell walls of transgenic tubers were mechanically stronger (Orfila et al., 2012). The generation and analysis of biomass crop plants overexpressing *PAE*s should indicate whether manipulating pectin acetylation levels will in fact enhance biomass for biofuel production. The accumulation of acetate in saccharified biomass, which is derived mainly from de-acetylation of xylans but also arises partly from pectin de-acetylation, can act as a potent inhibitor of biofuel conversion (Gille and Pauly, 2012), and the partial reduction of cell wall acetylation by modulating pectin acetyltransferase and/or acetylesterase activities might therefore improve microbial viability during fermentation and enhance the conversion efficiency of biomass to biofuel (**Figure 1B**).

Because of its crosslinking and water complexation properties, pectin is also a determinant of cell wall porosity (Willats et al., 2001). In one study, treatment with pectin-degrading enzymes such as endo-PGs increased wall pore size and the ability of larger molecules to pass through the wall (Baron-Epel et al., 1988); however, treatment with cellulysin or protease did not affect porosity, implying that pectin rather than cellulose is a major mediator of wall porosity. Wall porosity is also regulated by borate diester-coupled RG-II linkages (O’Neill et al., 1996; Fleischer et al., 1999). In the walls of pollen tubes, which have unique composition and mechanical properties, pectin influences both cell wall porosity and mechanical strength (Derksen et al., 2011). Because the average pore size in cell walls is similar to that of many globular proteins (Carpita et al., 1979), increased wall porosity should correlate with higher diffusion rates and accessibility to wall components for degradative enzymes during biomass processing. A relatively unexplored idea is the extent to which the aforementioned effects of pectin on wall rigidity might influence the physical properties of biomass during pretreatment. Conceivably, stiffening cell walls by the manipulation of Ca^2^^+^-mediated pectin crosslinks might enhance the fracturability of biomass, but experimental support for this idea is currently lacking.

## BIOFUELS FROM PECTIN-RICH FEEDSTOCKS

Although lignocellulosic biofuels are a promising renewable energy resource, the recalcitrance of biomass to degradation presents a major roadblock to their production. To increase biofuel yields, one strategy is to improve the conversion efficiency of plant cell walls to bioethanol (Jordan et al., 2012). The conversion process can be simplified by altering lignocellulose composition in bioenergy crop plants through genetic and molecular engineering (Demura and Ye, 2010; Pauly and Keegstra, 2010). Another strategy is to exploit existing plants with large amounts of easily digestible biomass (Somerville et al., 2010). At present, bioethanol is mainly produced from corn in the United States (Jordan et al., 2012), where the government has set a goal to produce 30% of liquid transportation fuels from biomass by 2030 (Demura and Ye, 2010). Like starch, pectins are largely water-soluble and relatively easy to degrade in comparison to other wall components. Pectins are abundant in waste residues of fruits and vegetables, which could be used as feedstocks for ethanol production. These pectin-rich residues have in many cases already been pretreated or processed and contain low lignin levels, which should facilitate the deconstruction of their cell walls and reduce the usage of degradative enzymes (Edwards and Doran-Peterson, 2012). So far, several pectin-rich materials, including sugar beet pulp (Rorick et al., 2011), citrus waste (Lopez et al., 2010; Pourbafrani et al., 2010), and apple pomace (Canteri-Schemin et al., 2005) have been analyzed as bioenergy feedstocks. Recent research has also indicated that potato pulp is an attractive raw material for bioethanol production since it contains abundant polysaccharides (Lesiecki et al., 2012). The use of pectin-rich resources as bioenergy feedstocks will require saccharification and fermentation methods that are optimized for the suite of sugars they contain, and efforts are already underway to generate microbial bioprocessing strains tailored to these materials (Edwards et al., 2011).

## PECTIN AS A HIGH-VALUE BIOMASS CO-PRODUCT

As a natural complex polysaccharide, pectin plays important industrial roles in several fields. Its physical and chemical properties make it a valuable material in the food and pharmaceutical industries (May, 1990). As a food additive, pectin is mainly used as a gelling agent in jams, a thickening and stabilizing agent in drinks, and as a gelatin substitute in baked foods (Srivastava and Malviya, 2011). Recent work has shown that the field application of pectin-derived oligosaccharides (PDOs) improves the coloration and anthocyanin content of seedless grapes (Ochoa-Villarreal et al., 2011), and recombinant PME has been used to increase the hardness of fruit products and reduce the turbidity of fruit juices (Jiang et al., 2012b).

Pectin is part of the soluble dietary fiber that exists in all fruits and vegetables and is thus beneficial for human health. Pectin consumption has been demonstrated to reduce blood cholesterol levels in humans, although the pectins used in these studies were administered at high doses and were not precisely characterized (Brouns et al., 2012). Modified citrus pectin (MCP) has been shown to enhance the immune system’s ability to prevent metastasis (Hurd, 1999) and inhibit cancer cell growth (Nangia-Makker et al., 2002; Jackson et al., 2007; Yan and Katz, 2010; Maxwell et al., 2012). The MCP functions synergistically with other compounds in inhibiting cancer cell growth (Jiang et al., 2012a), which is a promising result for the development of anti-metastatic drugs (Glinsky and Raz, 2009). Specifically, the RG-I component of pectin might contribute to its anticancer activity (Cheng et al., 2012). Because of its structural malleability, biodegradability, and tunable porosity, pectin is also used as a surface modifier for medical devices (Morra et al., 2004) and a material for biomedical applications including drug delivery, gene delivery, and tissue engineering (Munarin et al., 2011, 2012). These applications make pectin, either in its unmodified or derivatized forms, a potentially high-value component of biomass (**Figure 1C**).

## CONCLUSION

Pectins are one of the most structurally complex classes of molecules in nature, and it is perhaps due to this complexity that they serve a multitude of functions during plant growth and development. Depending on the feedstock, processing regime, and desired end products, pectin can be viewed either as a hindrance to biomass degradability, a source of fermentable sugars in its own right, or a potentially valuable co-product of biofuel production. A more comprehensive understanding of pectin structure and the mechanisms of its synthesis, modification, and degradation will allow for the enhancement of efforts to grow and utilize plants as renewable sources of food, materials, and energy.

## Conflict of Interest Statement

The authors declare that the research was conducted in the absence of any commercial or financial relationships that could be construed as a potential conflict of interest.
